# 6-Butyryl-5-hy­droxy-4-phenyl­seselin

**DOI:** 10.1107/S160053681003415X

**Published:** 2010-08-28

**Authors:** Thammarat Aree, Santi Tip-pyang, Preecha Sowanthip

**Affiliations:** a Department of Chemistry, Faculty of Science, Chulalongkorn University, Phyathai Road, Pathumwan, Bangkok 10330, Thailand

## Abstract

In the title coumarin compound (systematic name: 6-butyryl-5-hy­droxy-8,8-dimethyl-4-phenyl-2*H*,8*H*-benzo[1,2-*b*;3,4-*b*′]dipyran-2-one), C_24_H_22_O_5_, also known as mammea A/AC cyclo D, the chromene and pyran rings are almost coplanar with a maximum deviation from the mean plane of 0.295 (2) Å. The attached phenyl group is inclined at 53.49 (8)° with respect to the chromene ring. The mol­ecular structure is stabilized by an intra­molecular O—H⋯O hydrogen bond. In the crystal, mol­ecules are linked into sheets parallel to (101) by inter­molecular C—H⋯O hydrogen bonds. Adjacent sheets are sustained by inter­molecular C—H⋯π and π–π [centroid–centroid distance = 4.471 (2) Å] inter­actions.

## Related literature

For the structural characterization of mammea A/AC cyclo D, see: Thebtaranonth *et al.* (1981 ▶); Morel *et al.* (1999 ▶); Kaweetripob *et al.* (2000 ▶). For its anti-HIV activity, see: Márquez *et al.* (2005 ▶); Bedoya *et al.* (2005 ▶) and for its anti­cancer activity, see: Reyes-Chilpa *et al.* (2004 ▶). For related coumarins, see: Mahidol *et al.* (2002 ▶). For a review on the cytotoxic activity of coumarins, see: Kostova (2005 ▶).
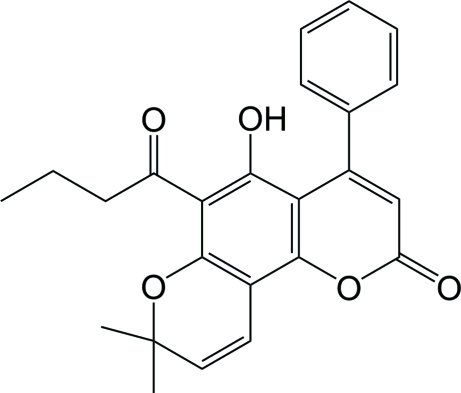


## Experimental

### 

#### Crystal data

C_24_H_22_O_5_
*M* *_r_* = 390.42
Monoclinic, 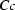

*a* = 17.0746 (4) Å
*b* = 13.4170 (4) Å
*c* = 8.7607 (3) Å
β = 90.341 (1)°
*V* = 2006.95 (10) Å^3^
*Z* = 4
Mo *K*α radiation
μ = 0.09 mm^−1^
*T* = 298 K
0.40 × 0.32 × 0.16 mm

#### Data collection

Bruker SMART APEXII CCD area-detector diffractometer
Absorption correction: multi-scan (*SADABS*; Bruker, 2005 ▶) *T*_min_ = 0.965, *T*_max_ = 0.986
5484 measured reflections
2115 independent reflections
1714 reflections with *I* > 2σ(*I*)
*R*_int_ = 0.023

#### Refinement

*R*[*F* ^2^ > 2σ(*F* ^2^)] = 0.037
*wR*(*F* ^2^) = 0.100
*S* = 1.03
2115 reflections
265 parameters
2 restraints
H-atom parameters constrained
Δρ_max_ = 0.13 e Å^−3^
Δρ_min_ = −0.14 e Å^−3^

### 

Data collection: *APEX2* (Bruker, 2005 ▶); cell refinement: *SAINT* (Bruker, 2005 ▶); data reduction: *SAINT*; program(s) used to solve structure: *SHELXTL* (Sheldrick, 2008 ▶); program(s) used to refine structure: *SHELXTL*; molecular graphics: *Mercury* (Macrae *et al.* 2006 ▶); software used to prepare material for publication: *SHELXTL*.

## Supplementary Material

Crystal structure: contains datablocks I, global. DOI: 10.1107/S160053681003415X/ds2051sup1.cif

Structure factors: contains datablocks I. DOI: 10.1107/S160053681003415X/ds2051Isup2.hkl

Additional supplementary materials:  crystallographic information; 3D view; checkCIF report

## Figures and Tables

**Table 1:** Hydrogen-bond geometry (Å, °) *Cg*1 is the centroid of the C1′–C6′ring.

*D*—H⋯*A*	*D*—H	H⋯*A*	*D*⋯*A*	*D*—H⋯*A*
O3—H3*O*⋯O1"′	0.82	1.73	2.464 (3)	149
C3"′—H32⋯O2^i^	0.97	2.71	3.522 (5)	142
C4′—H4′⋯O2^ii^	0.93	2.70	3.396 (4)	132
C6"—H61"⋯*Cg*1^iii^	0.96	2.75	3.646 (4)	156

Symmetry codes: (i) 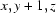; (ii) 
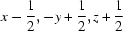
; (iii) 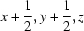.

**Fig. 1.**
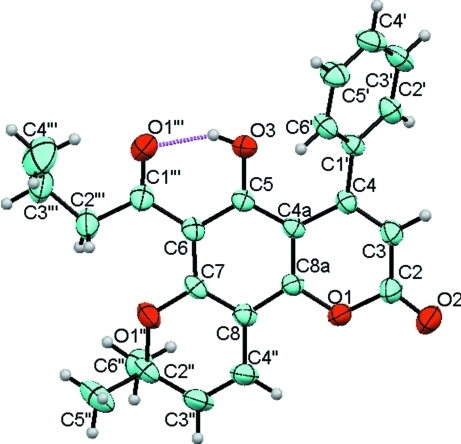
The molecular structure of the title compound, with atomic numbering scheme and 40% probability displacement ellipsoids.

**Fig. 2.**
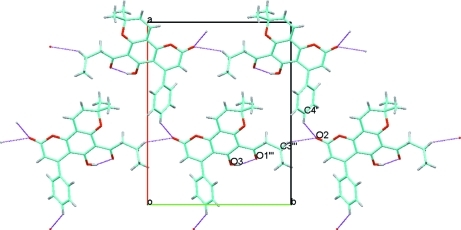
An infinite sheet parallel to (101) formed by intermolecular C—H···O hydrogen bonds (dotted lines).

**Fig. 3.**
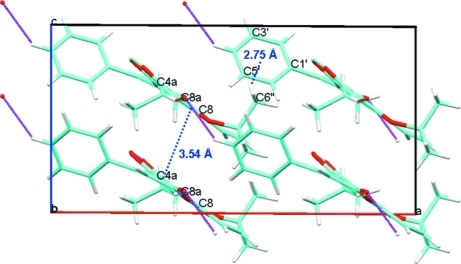
Parallel, infinite sheets are sustained by intermolecular C—H···π and π–π interactions.

## supplementary crystallographic information

### Comment

The title coumarin compound, 6-butyryl-5-hydroxy-4-phenylseselin or mammea A/AC cyclo D (Fig. 1) was isolated from the hexane crude extract of the flowers of *Mammea siamensis* (*Sarapee* in Thai). Several coumarins derived from the same flowers have been reported, see for example Mahidol *et al.* 2002 and other references cited therein. In this work, we report the crystal structure of mammea A/AC cyclo D.

The molecular structure consists of one chromene ring, one pyran ring and one phenyl ring (Fig. 1). The chromene and pyran rings are almost coplanar. Atoms C2, C2", C4" and O1" most deviate from the mean plane by 0.133 (3), 0.295 (2), –0.154 (3) and –0.172 (2) Å, respectively. The butyraldehyde group, hydroxy group and atom O2 displace from the chromene plane to greater extents: 0.326 (3) Å, O3; 0.307 (4) Å, O1"'; and –0.303 (7) Å, C3"'. The methyl C4"', C5" and C6" atoms point upwards and downwards the chromene ring with torsion angles of –73.8 (6)° for C1"'—C2"'—C3"'—C4"', –142.8 (3)° for C4"—C3"—C2"—C5" and 91.7 (4)° for C4"—C3"—C2"—C6". The attached phenyl group inclines by 53.49 (8)° against the chromene ring. The molecular structure is stabilized by intramolecular O3—H···O1"' hydrogen bond.

In the crystal, the molecules are linked into sheets parallel to (101) by intermolecular, bifurcated C3"'—H32···O2(*x*, *y* + 1, *z*) and C4'—H4'···O2(*x* – 0.5, –*y* + 0.5, *z* + 0.5) hydrogen bonds (Fig. 2 and Table 1). The adjacent sheets are sustained by intermolecular C6"—H61"···π (ring C1'—C2'—C3'—C4'—C5'—C6') and π–π (two adjacent rings of C4a—C5—C6—C7—C8—C8a) interactions (Fig. 3). The corresponding distance from atom H to the phenyl-ring center is 2.75 Å and the interplanar spacing is 3.54 Å.

### Experimental

The title coumarin compound was isolated from the hexane crude extract of the flowers of *Mammea siamensis*, which is a Thai medicinal plant, locally known as *Sarapee*. This coumarin mammea A/AC cyclo D was known for almost 30 years. Its structure was ambigously characterized by spectroscopic techniques (Thebtaranonth *et al.*, 1981; Morel *et al.*, 1999; and Kaweetripob *et al.*, 2000). Other coumarins were also isolated from the same flower (Mahidol *et al.*, 2002 and other references cited therein).

The light yellow, block-like single crystals were obtained by slow evaporation of a hexane–dichloromethane solution at room temperature.

### Refinement

All H atoms were located in a difference Fourier map and then refined using a riding model: C–H = 0.97 Å(secondary), 0.93 Å (aromatic), 0.96 Å (methyl), O–H = 0.82 Å (hydroxy), and *U*_iso_(H) = 1.2*U*_eq_(C,O) and *U*_iso_(H) = 1.5*U*_eq_(methyl C). In the absence of significant anomalous scattering effects, Friedel pairs were averaged and therefore, the absolute structure could not be determined.

### Crystal data

**Table:**

C_24_H_22_O_5_	*F*(000) = 824
*M**_r_* = 390.42	*D*_x_ = 1.292 Mg m^−^^3^
Monoclinic, *C**c*	Melting point = 412–413 K
Hall symbol: C -2yc	Mo *K*α radiation, λ = 0.71073 Å
*a* = 17.0746 (4) Å	Cell parameters from 2083 reflections
*b* = 13.4170 (4) Å	θ = 2.4–24.3°
*c* = 8.7607 (3) Å	µ = 0.09 mm^−^^1^
β = 90.341 (1)°	*T* = 298 K
*V* = 2006.95 (10) Å^3^	Block, light yellow
*Z* = 4	0.40 × 0.32 × 0.16 mm

### Data collection

**Table:**

Bruker SMART APEXII CCD area-detector diffractometer	2115 independent reflections
Radiation source: fine-focus sealed tube	1714 reflections with *I* > 2σ(*I*)
graphite	*R*_int_ = 0.023
φ and ω scans	θ_max_ = 26.7°, θ_min_ = 1.9°
Absorption correction: multi-scan (*SADABS*; Bruker, 2005)	*h* = −21→21
*T*_min_ = 0.965, *T*_max_ = 0.986	*k* = −16→12
5484 measured reflections	*l* = −11→10

### Refinement

**Table:**

Refinement on *F*^2^	Primary atom site location: structure-invariant direct methods
Least-squares matrix: full	Secondary atom site location: difference Fourier map
*R*[*F*^2^ > 2σ(*F*^2^)] = 0.037	Hydrogen site location: inferred from neighbouring sites
*wR*(*F*^2^) = 0.100	H-atom parameters constrained
*S* = 1.03	*w* = 1/[σ^2^(*F*_o_^2^) + (0.0539*P*)^2^ + 0.3001*P*] where *P* = (*F*_o_^2^ + 2*F*_c_^2^)/3
2115 reflections	(Δ/σ)_max_ < 0.001
265 parameters	Δρ_max_ = 0.13 e Å^−^^3^
2 restraints	Δρ_min_ = −0.14 e Å^−^^3^

### Special details

**Table:**

Geometry. All e.s.d.'s (except the e.s.d. in the dihedral angle between two l.s. planes) are estimated using the full covariance matrix. The cell e.s.d.'s are taken into account individually in the estimation of e.s.d.'s in distances, angles and torsion angles; correlations between e.s.d.'s in cell parameters are only used when they are defined by crystal symmetry. An approximate (isotropic) treatment of cell e.s.d.'s is used for estimating e.s.d.'s involving l.s. planes.
Refinement. Refinement of *F*^2^ against ALL reflections. The weighted *R*-factor *wR* and goodness of fit *S* are based on *F*^2^, conventional *R*-factors *R* are based on *F*, with *F* set to zero for negative *F*^2^. The threshold expression of *F*^2^ > σ(*F*^2^) is used only for calculating *R*-factors(gt) *etc*. and is not relevant to the choice of reflections for refinement. *R*-factors based on *F*^2^ are statistically about twice as large as those based on *F*, and *R*- factors based on ALL data will be even larger.

### Fractional atomic coordinates and isotropic or equivalent isotropic displacement parameters (Å^2^)

**Table:**

	*x*	*y*	*z*	*U*_iso_*/*U*_eq_	
O1	0.37812 (12)	0.33800 (15)	0.0704 (2)	0.0539 (5)	
C2	0.33746 (18)	0.2581 (2)	0.1325 (4)	0.0585 (8)	
C3	0.26471 (17)	0.2825 (2)	0.2042 (4)	0.0527 (7)	
H3	0.2335	0.2304	0.2382	0.063*	
C4	0.23882 (15)	0.3763 (2)	0.2253 (3)	0.0417 (6)	
C4A	0.28947 (13)	0.4585 (2)	0.1797 (3)	0.0381 (6)	
C5	0.27705 (14)	0.5596 (2)	0.2138 (3)	0.0401 (6)	
C6	0.32418 (15)	0.6360 (2)	0.1496 (3)	0.0418 (6)	
C7	0.38818 (14)	0.6053 (2)	0.0584 (3)	0.0430 (6)	
C8	0.40587 (14)	0.5060 (2)	0.0336 (3)	0.0426 (6)	
C8A	0.35629 (14)	0.4350 (2)	0.0958 (3)	0.0413 (6)	
O2	0.36629 (16)	0.17743 (18)	0.1167 (4)	0.0866 (8)	
O3	0.22095 (11)	0.58102 (16)	0.3131 (2)	0.0545 (5)	
H3O	0.2187	0.6415	0.3254	0.065*	
C1'	0.15739 (15)	0.3906 (2)	0.2792 (3)	0.0423 (6)	
C2'	0.12989 (18)	0.3393 (2)	0.4053 (3)	0.0517 (7)	
H2'	0.1639	0.2998	0.4623	0.062*	
C3'	0.0518 (2)	0.3467 (3)	0.4467 (3)	0.0634 (9)	
H3'	0.0335	0.3115	0.5307	0.076*	
C4'	0.00155 (18)	0.4058 (3)	0.3643 (4)	0.0651 (9)	
H4'	−0.0505	0.4115	0.3936	0.078*	
C5'	0.02778 (17)	0.4563 (2)	0.2394 (4)	0.0613 (8)	
H5'	−0.0066	0.4958	0.1831	0.074*	
C6'	0.10527 (16)	0.4491 (2)	0.1961 (3)	0.0518 (7)	
H6'	0.1227	0.4836	0.1107	0.062*	
O1"	0.43245 (11)	0.67654 (16)	−0.0081 (2)	0.0583 (6)	
C2"	0.51443 (16)	0.6551 (2)	−0.0456 (3)	0.0512 (7)	
C3"	0.52184 (19)	0.5511 (3)	−0.1026 (4)	0.0659 (9)	
H3"	0.5624	0.5355	−0.1689	0.079*	
C4"	0.47252 (17)	0.4806 (3)	−0.0619 (3)	0.0590 (8)	
H4"	0.4802	0.4152	−0.0938	0.071*	
C5"	0.5349 (2)	0.7323 (4)	−0.1653 (5)	0.0883 (13)	
H51"	0.5019	0.7234	−0.2533	0.132*	
H53"	0.5887	0.7246	−0.1941	0.132*	
H52"	0.5271	0.7979	−0.1242	0.132*	
C6"	0.56228 (19)	0.6696 (3)	0.0983 (4)	0.0650 (9)	
H61"	0.5531	0.7352	0.1383	0.098*	
H62"	0.6169	0.6621	0.0754	0.098*	
H63"	0.5473	0.6207	0.1726	0.098*	
O1"'	0.25223 (16)	0.75871 (17)	0.2771 (3)	0.0754 (7)	
C1"'	0.30374 (18)	0.7407 (2)	0.1820 (4)	0.0534 (7)	
C2"'	0.3419 (2)	0.8263 (2)	0.1013 (5)	0.0707 (9)	
H21	0.3395	0.8142	−0.0078	0.085*	
H22	0.3968	0.8285	0.1306	0.085*	
C3"'	0.3056 (3)	0.9268 (3)	0.1337 (8)	0.1047 (16)	
H31	0.2988	0.9338	0.2430	0.126*	
H32	0.3413	0.9786	0.1007	0.126*	
C4"'	0.2279 (3)	0.9421 (4)	0.0562 (8)	0.131 (2)	
H41	0.2350	0.9428	−0.0524	0.197*	
H42	0.2059	1.0045	0.0882	0.197*	
H43	0.1931	0.8889	0.0832	0.197*	

### Atomic displacement parameters (Å^2^)

**Table:**

	*U*^11^	*U*^22^	*U*^33^	*U*^12^	*U*^13^	*U*^23^
O1	0.0436 (10)	0.0447 (11)	0.0734 (13)	−0.0010 (8)	0.0108 (9)	−0.0126 (9)
C2	0.0485 (16)	0.0413 (17)	0.086 (2)	−0.0067 (13)	0.0061 (16)	−0.0075 (15)
C3	0.0431 (15)	0.0435 (15)	0.0716 (19)	−0.0084 (12)	0.0022 (13)	0.0007 (14)
C4	0.0370 (13)	0.0426 (14)	0.0456 (15)	−0.0053 (11)	−0.0006 (11)	0.0026 (11)
C4A	0.0330 (13)	0.0428 (14)	0.0386 (13)	−0.0045 (11)	−0.0004 (10)	−0.0006 (11)
C5	0.0347 (12)	0.0447 (15)	0.0410 (13)	−0.0012 (11)	−0.0023 (10)	−0.0002 (12)
C6	0.0329 (12)	0.0441 (15)	0.0482 (14)	−0.0034 (11)	−0.0063 (10)	0.0018 (11)
C7	0.0322 (12)	0.0518 (16)	0.0450 (14)	−0.0107 (11)	−0.0031 (10)	0.0063 (12)
C8	0.0368 (12)	0.0485 (15)	0.0426 (13)	−0.0060 (11)	0.0001 (10)	−0.0046 (12)
C8A	0.0357 (14)	0.0437 (15)	0.0444 (14)	−0.0024 (11)	0.0000 (11)	−0.0062 (11)
O2	0.0718 (15)	0.0470 (14)	0.141 (2)	0.0045 (12)	0.0231 (15)	−0.0142 (15)
O3	0.0476 (11)	0.0509 (12)	0.0652 (13)	−0.0020 (9)	0.0161 (9)	−0.0061 (10)
C1'	0.0368 (13)	0.0457 (15)	0.0445 (13)	−0.0097 (11)	0.0020 (10)	−0.0001 (12)
C2'	0.0556 (17)	0.0551 (18)	0.0443 (15)	−0.0095 (14)	−0.0012 (12)	0.0048 (13)
C3'	0.0620 (19)	0.082 (2)	0.0466 (17)	−0.0215 (17)	0.0152 (14)	0.0053 (16)
C4'	0.0436 (16)	0.083 (2)	0.069 (2)	−0.0096 (15)	0.0154 (14)	−0.0080 (18)
C5'	0.0419 (15)	0.071 (2)	0.071 (2)	0.0020 (14)	−0.0005 (14)	0.0055 (16)
C6'	0.0408 (14)	0.0583 (18)	0.0564 (16)	−0.0082 (12)	0.0040 (11)	0.0115 (13)
O1"	0.0438 (11)	0.0547 (13)	0.0765 (14)	−0.0081 (9)	0.0076 (10)	0.0172 (10)
C2"	0.0376 (14)	0.0625 (18)	0.0537 (17)	−0.0125 (12)	0.0047 (11)	0.0103 (14)
C3"	0.0513 (17)	0.086 (3)	0.0604 (18)	−0.0193 (16)	0.0220 (14)	−0.0193 (17)
C4"	0.0473 (16)	0.066 (2)	0.0637 (19)	−0.0107 (15)	0.0178 (13)	−0.0189 (16)
C5"	0.059 (2)	0.117 (3)	0.089 (3)	−0.016 (2)	0.0098 (18)	0.045 (2)
C6"	0.0623 (19)	0.066 (2)	0.067 (2)	−0.0040 (15)	−0.0097 (15)	−0.0067 (16)
O1"'	0.0721 (15)	0.0505 (13)	0.1036 (19)	0.0059 (11)	0.0179 (14)	−0.0090 (12)
C1"'	0.0393 (14)	0.0468 (16)	0.0739 (19)	−0.0003 (12)	−0.0069 (14)	0.0006 (15)
C2"'	0.0588 (19)	0.0437 (18)	0.110 (3)	−0.0058 (14)	−0.0009 (18)	0.0087 (18)
C3"'	0.089 (3)	0.047 (2)	0.178 (5)	0.001 (2)	0.006 (3)	0.011 (3)
C4"'	0.107 (4)	0.086 (4)	0.200 (6)	0.037 (3)	−0.002 (4)	0.023 (4)

### Geometric parameters (Å, °)

**Table:**

O1—C8A	1.372 (3)	C5'—C6'	1.382 (4)
O1—C2	1.389 (4)	C5'—H5'	0.9300
C2—O2	1.198 (4)	C6'—H6'	0.9300
C2—C3	1.433 (4)	O1"—C2"	1.468 (3)
C3—C4	1.347 (4)	C2"—C3"	1.488 (5)
C3—H3	0.9300	C2"—C6"	1.510 (4)
C4—C4A	1.458 (3)	C2"—C5"	1.516 (5)
C4—C1'	1.483 (4)	C3"—C4"	1.317 (4)
C4A—C8A	1.397 (3)	C3"—H3"	0.9300
C4A—C5	1.406 (3)	C4"—H4"	0.9300
C5—O3	1.329 (3)	C5"—H51"	0.9600
C5—C6	1.421 (4)	C5"—H53"	0.9600
C6—C7	1.418 (4)	C5"—H52"	0.9600
C6—C1"'	1.476 (4)	C6"—H61"	0.9600
C7—O1"	1.353 (3)	C6"—H62"	0.9600
C7—C8	1.385 (4)	C6"—H63"	0.9600
C8—C8A	1.388 (4)	O1"'—C1"'	1.239 (4)
C8—C4"	1.457 (4)	C1"'—C2"'	1.500 (5)
O3—H3O	0.8200	C2"'—C3"'	1.511 (6)
C1'—C2'	1.385 (4)	C2"'—H21	0.9700
C1'—C6'	1.389 (4)	C2"'—H22	0.9700
C2'—C3'	1.387 (4)	C3"'—C4"'	1.501 (7)
C2'—H2'	0.9300	C3"'—H31	0.9700
C3'—C4'	1.371 (5)	C3"'—H32	0.9700
C3'—H3'	0.9300	C4"'—H41	0.9600
C4'—C5'	1.365 (4)	C4"'—H42	0.9600
C4'—H4'	0.9300	C4"'—H43	0.9600
			
C8A—O1—C2	122.1 (2)	C7—O1"—C2"	119.6 (2)
O2—C2—O1	116.5 (3)	O1"—C2"—C3"	110.0 (2)
O2—C2—C3	127.9 (3)	O1"—C2"—C6"	107.5 (2)
O1—C2—C3	115.6 (3)	C3"—C2"—C6"	110.7 (3)
C4—C3—C2	124.1 (3)	O1"—C2"—C5"	104.2 (3)
C4—C3—H3	118.0	C3"—C2"—C5"	112.8 (3)
C2—C3—H3	118.0	C6"—C2"—C5"	111.3 (3)
C3—C4—C4A	118.2 (2)	C4"—C3"—C2"	121.8 (3)
C3—C4—C1'	118.3 (2)	C4"—C3"—H3"	119.1
C4A—C4—C1'	123.2 (2)	C2"—C3"—H3"	119.1
C8A—C4A—C5	117.0 (2)	C3"—C4"—C8	119.4 (3)
C8A—C4A—C4	117.5 (2)	C3"—C4"—H4"	120.3
C5—C4A—C4	125.5 (2)	C8—C4"—H4"	120.3
O3—C5—C4A	117.2 (2)	C2"—C5"—H51"	109.5
O3—C5—C6	121.0 (2)	C2"—C5"—H53"	109.5
C4A—C5—C6	121.7 (2)	H51"—C5"—H53"	109.5
C7—C6—C5	117.0 (2)	C2"—C5"—H52"	109.5
C7—C6—C1"'	124.6 (3)	H51"—C5"—H52"	109.5
C5—C6—C1"'	118.4 (3)	H53"—C5"—H52"	109.5
O1"—C7—C8	119.3 (2)	C2"—C6"—H61"	109.5
O1"—C7—C6	118.2 (2)	C2"—C6"—H62"	109.5
C8—C7—C6	122.5 (2)	H61"—C6"—H62"	109.5
C7—C8—C8A	117.7 (2)	C2"—C6"—H63"	109.5
C7—C8—C4"	119.1 (3)	H61"—C6"—H63"	109.5
C8A—C8—C4"	123.1 (3)	H62"—C6"—H63"	109.5
O1—C8A—C8	114.8 (2)	O1"'—C1"'—C6	119.0 (3)
O1—C8A—C4A	121.5 (2)	O1"'—C1"'—C2"'	118.7 (3)
C8—C8A—C4A	123.7 (2)	C6—C1"'—C2"'	122.3 (3)
C5—O3—H3O	109.5	C1"'—C2"'—C3"'	114.5 (3)
C2'—C1'—C6'	118.6 (2)	C1"'—C2"'—H21	108.6
C2'—C1'—C4	120.8 (3)	C3"'—C2"'—H21	108.6
C6'—C1'—C4	120.3 (2)	C1"'—C2"'—H22	108.6
C1'—C2'—C3'	120.2 (3)	C3"'—C2"'—H22	108.6
C1'—C2'—H2'	119.9	H21—C2"'—H22	107.6
C3'—C2'—H2'	119.9	C4"'—C3"'—C2"'	113.6 (4)
C4'—C3'—C2'	120.2 (3)	C4"'—C3"'—H31	108.9
C4'—C3'—H3'	119.9	C2"'—C3"'—H31	108.9
C2'—C3'—H3'	119.9	C4"'—C3"'—H32	108.9
C5'—C4'—C3'	120.1 (3)	C2"'—C3"'—H32	108.9
C5'—C4'—H4'	119.9	H31—C3"'—H32	107.7
C3'—C4'—H4'	119.9	C3"'—C4"'—H41	109.5
C4'—C5'—C6'	120.3 (3)	C3"'—C4"'—H42	109.5
C4'—C5'—H5'	119.9	H41—C4"'—H42	109.5
C6'—C5'—H5'	119.9	C3"'—C4"'—H43	109.5
C5'—C6'—C1'	120.5 (3)	H41—C4"'—H43	109.5
C5'—C6'—H6'	119.8	H42—C4"'—H43	109.5
C1'—C6'—H6'	119.8		
			
C8A—O1—C2—O2	−172.2 (3)	C4—C4A—C8A—O1	−6.5 (3)
C8A—O1—C2—C3	9.4 (4)	C5—C4A—C8A—C8	−6.3 (3)
O2—C2—C3—C4	175.7 (4)	C4—C4A—C8A—C8	174.1 (2)
O1—C2—C3—C4	−6.1 (5)	C3—C4—C1'—C2'	50.3 (4)
C2—C3—C4—C4A	−3.2 (4)	C4A—C4—C1'—C2'	−136.1 (3)
C2—C3—C4—C1'	170.7 (3)	C3—C4—C1'—C6'	−124.1 (3)
C3—C4—C4A—C8A	9.5 (3)	C4A—C4—C1'—C6'	49.6 (4)
C1'—C4—C4A—C8A	−164.1 (2)	C6'—C1'—C2'—C3'	0.0 (4)
C3—C4—C4A—C5	−170.0 (2)	C4—C1'—C2'—C3'	−174.5 (3)
C1'—C4—C4A—C5	16.3 (4)	C1'—C2'—C3'—C4'	−0.8 (5)
C8A—C4A—C5—O3	−169.6 (2)	C2'—C3'—C4'—C5'	1.1 (5)
C4—C4A—C5—O3	9.9 (3)	C3'—C4'—C5'—C6'	−0.7 (5)
C8A—C4A—C5—C6	8.1 (3)	C4'—C5'—C6'—C1'	−0.2 (5)
C4—C4A—C5—C6	−172.4 (2)	C2'—C1'—C6'—C5'	0.5 (4)
O3—C5—C6—C7	173.0 (2)	C4—C1'—C6'—C5'	175.0 (3)
C4A—C5—C6—C7	−4.6 (3)	C8—C7—O1"—C2"	−27.2 (3)
O3—C5—C6—C1"'	−6.8 (3)	C6—C7—O1"—C2"	153.9 (2)
C4A—C5—C6—C1"'	175.5 (2)	C7—O1"—C2"—C3"	38.7 (3)
C5—C6—C7—O1"	177.9 (2)	C7—O1"—C2"—C6"	−81.9 (3)
C1"'—C6—C7—O1"	−2.3 (4)	C7—O1"—C2"—C5"	159.9 (3)
C5—C6—C7—C8	−1.1 (3)	O1"—C2"—C3"—C4"	−27.0 (4)
C1"'—C6—C7—C8	178.8 (2)	C6"—C2"—C3"—C4"	91.7 (4)
O1"—C7—C8—C8A	−176.1 (2)	C5"—C2"—C3"—C4"	−142.8 (3)
C6—C7—C8—C8A	2.9 (4)	C2"—C3"—C4"—C8	4.1 (5)
O1"—C7—C8—C4"	0.9 (4)	C7—C8—C4"—C3"	10.7 (4)
C6—C7—C8—C4"	179.8 (2)	C8A—C8—C4"—C3"	−172.5 (3)
C2—O1—C8A—C8	176.2 (3)	C7—C6—C1"'—O1"'	−171.8 (3)
C2—O1—C8A—C4A	−3.2 (4)	C5—C6—C1"'—O1"'	8.1 (4)
C7—C8—C8A—O1	−178.4 (2)	C7—C6—C1"'—C2"'	9.6 (4)
C4"—C8—C8A—O1	4.7 (3)	C5—C6—C1"'—C2"'	−170.5 (3)
C7—C8—C8A—C4A	0.9 (4)	O1"'—C1"'—C2"'—C3"'	−6.7 (5)
C4"—C8—C8A—C4A	−175.9 (2)	C6—C1"'—C2"'—C3"'	171.9 (3)
C5—C4A—C8A—O1	173.1 (2)	C1"'—C2"'—C3"'—C4"'	−73.8 (6)

### Hydrogen-bond geometry (Å, °)

**Table:**

Cg1 is the centroid of the C1'–C6'ring.

**Table:**

*D*—H···*A*	*D*—H	H···*A*	*D*···*A*	*D*—H···*A*
O3—H3O···O1"'	0.82	1.73	2.464 (3)	149.
C3"'—H32···O2^i^	0.97	2.71	3.522 (5)	142.
C4'—H4'···O2^ii^	0.93	2.70	3.396 (4)	132.
C6"—H61"···Cg1^iii^	0.96	2.75	3.646 (4)	156

Symmetry codes: (i) *x*, *y*+1, *z*; (ii) *x*−1/2, −*y*+1/2, *z*+1/2; (iii) *x*+1/2, *y*+1/2, *z*.
